# Poly(ethylene oxide)-Based Copolymer-IL Composite Membranes for CO_2_ Separation

**DOI:** 10.3390/membranes13010026

**Published:** 2022-12-25

**Authors:** Dionysios Vroulias, Eirini Staurianou, Theophilos Ioannides, Valadoula Deimede

**Affiliations:** 1 Department of Chemistry, University of Patras, GR-26504 Patras, Greece; 2 Foundation for Research and Technology-Hellas, Institute of Chemical Engineering Sciences (FORTH/ICE-HT), GR-26504 Patras, Greece

**Keywords:** PEO-based copolymers, polymer-IL composite membranes, CO_2_ permeability, water vapor permeability, CO_2_/CH_4_ selectivity, CO_2_/H_2_ selectivity

## Abstract

Poly(ethylene oxide) (PEO)-based copolymers are at the forefront of advanced membrane materials for selective CO_2_ separation. In this work, free-standing composite membranes were prepared by blending imidazolium-based ionic liquids (ILs) having different structural characteristics with a PEO-based copolymer previously developed by our group, targeting CO_2_ permeability improvement and effective CO_2_/gas separation. The effect of IL loading (30 and 40 wt%), alkyl chain length of the imidazolium cation (ethyl- and hexyl- chain) and the nature of the anion (TFSI^-^, C(CN)_3_^-^) on physicochemical and gas transport properties were studied. Among all composite membranes, PEO-based copolymer with 40 wt% IL3-[HMIM][TFSI] containing the longer alkyl chain of the cation and TFSI^-^ as the anion exhibited the highest CO_2_ permeability of 46.1 Barrer and ideal CO_2_/H_2_ and CO_2_/CH_4_ selectivities of 5.6 and 39.0, respectively, at 30 °C. In addition, almost all composite membranes surpassed the upper bound limit for CO_2_/H_2_ separation. The above membrane showed the highest water vapor permeability value of 50,000 Barrer under both wet and dry conditions and a corresponding H_2_O/CO_2_ ideal selectivity value of 1080; values that are comparable with those reported for other highly water-selective PEO-based polymers. These results suggest the potential application of this membrane in hydrogen purification and dehydration of CO_2_ gas streams.

## 1. Introduction

The increase of carbon dioxide (CO_2_) concentration in the atmosphere due to anthropogenic activities such as burning of fossil fuels (i.e., coal, oil and natural gas), deforestation and wildfires is the main cause of global warming and climate change [1]. Carbon capture and storage (CCS) is an approach for reducing CO_2_ emissions by removing CO_2_ from large industrial and power plants, followed by its compression, transport and permanent storage [2]. Removal of CO_2_ from hydrogen streams produced by the steam reforming process and water gas shift reaction can also be viewed in a similar context. CO_2_ separation using polymeric membranes is a promising technology and has many advantages over other methods, namely absorption, pressure swing adsorption and cryogenic distillation [3]. The advantages include low energy consumption, simple installation and scale-up and eco-friendly characteristics [4,5].

Copolymer and polymeric ionic liquid (PIL)-based membranes containing poly(ethylene oxide) (PEO) units, such as Pebax^®^ (poly(amide-b-ethylene oxide)), Polyactive^TM^ (PEO_75_PBT_25_), PEGMA-950_2_ and PIL_750_-MeSO_4_, have shown improved CO_2_ permeation properties with increasing PEO weight content owing to the higher contribution of polar ethylene oxide (EO) units in the system, which interact favorably with CO_2_ [6,7,8,9]. Considering the high CO_2_ affinity of PEO groups due to the quadrupole-dipole intermolecular forces between the ether oxygen of PEO (C-O-C) and CO_2_ molecules, PEO-based membranes exhibit enhanced CO_2_ permeability and selectivity towards other gases [10,11]. However, it is well known that pure PEO suffers from poor film-forming properties and also has the tendency to crystallize, thereby leading to free volume reduction and deterioration of its CO_2_ separation performance. For this reason, our group has recently developed aromatic polyether copolymers bearing flexible, amorphous PEO pendants with excellent mechanical properties [8]. The copolymer membrane with the highest PEO content (Py-PEO_750_-49) exhibited the highest CO_2_ permeability (11.2 Barrer) with a corresponding maximum ideal CO_2_/H_2_ selectivity of 9.6, surpassing the upper bound. In order to further improve CO_2_ permeability, the latter was converted to its PIL analogue containing CO_2_-philic counter anions (C(CN)_3_^-^, TFSI^-^) [9]. However, the prepared PILs showed lower CO_2_ permeability values compared to that of the precursor due to their denser polymer chain packing, which hinders gas diffusion and thus results in a gas permeability decrease.

One different approach to enhancing CO_2_ transport through polymers is the incorporation of ionic liquids (ILs) into the polymer matrix [12,13,14,15,16]. In addition, such composite membranes show improved thermal and mechanical stability compared to supported ionic liquid membranes (SILMs) [16,17,18]. Ionic liquids act both as physical crosslinkers and plasticizers, thereby contributing to the reduction of polymer crystallinity of PEO-based membranes [19,20]. As a result, gas permeability increases, while ideal selectivity decreases following the “trade off” behavior [21,22].

PEO-based copolymer/IL membranes have been investigated as CO_2_-selective membranes, and the effect of PEO-based copolymer and IL nature, as well as IL loading, on gas permeation properties has also been studied. In specific, Bernardo et al. [20] prepared composite membranes by entrapping different amounts of [BMIM][CF_3_SO_3_] into two different, Pebax^®^1657 and Pebax^®^2533, matrices. In the less permeable Pebax^®^1657, the addition of IL resulted in a gas permeability increase and a slight ideal selectivity decrease for most gas pairs. However, for Pebax^®^2533, the gas permeation properties were not particularly affected by the presence of IL, suggesting that the polymer matrix plays a major role in gas permeation.

Regarding the effect of IL nature on gas separation performance, imidazolium-based ILs containing different alkyl chain length and glycine ([Gly]^-^) as a counter anion were incorporated into a Pebax^®^1657 matrix for the preparation of composite membranes [23]. A significant CO_2_ permeability and CO_2_/gas ideal selectivity enhancement was observed by increasing the alkyl chain length. The Pebax^®^1657/[C_6_MIM][Gly] membrane showed the best performance, exhibiting a CO_2_ permeability of 1490 Barrer and a corresponding CO_2_/N_2_ ideal selectivity of 95. On the other hand, it is well documented that anions dominate the interaction and affinity with CO_2_. For this reason, ILs with fluorinated anions such as [TFSI]^−^ [16,17,19,24,25,26,27,28,29], [TFO]^-^ [20,25], [BF_4_]^−^ [18,30] and [FAP]^−^ [25] are usually chosen due to their high CO_2_ affinity. For example, PolyActive^TM^ was blended with [BMIM][TFSI], and single and mixed gas transport properties of the prepared composite membranes were studied as a function of IL loading [31]. Upon addition of the IL, the crystallinity of the polyether phase was reduced and, as a result, gas diffusion and permeability were increased. The CO_2_/gas separation factors were close to ideal selectivity and remained unaffected at pressures up to 6 bar.

Aromatic polyethers that bear main chain, 2,6-substituted pyridine segments and short side chain PEO groups were already reported for CO_2_ separation by our group [8]. Despite their low CO_2_ permeability, these copolymers exhibit many advantageous characteristics, as they combine the excellent film-forming ability and thermomechanical stability with the high PEO content and amorphicity. Similar copolymer structures containing short side PEO segments have been synthesized and investigated as polymer electrolytes in lithium-ion batteries [32]. Therefore, in an attempt to improve both CO_2_ permeability and selectivity of the neat copolymer, it was blended with ionic liquids with different structural characteristics to afford composite membranes. Imidazolium-based ionic liquids containing CO_2_-philic anions were chosen to be incorporated into the polymer matrix, owing to their high CO_2_ solubility, as already reported [16,18]. In addition, it should be stressed that the presence of polar pyridine moieties along with sulfone groups in the polyether backbone enables the formation of hydrogen bonds with water, thereby promoting water vapor permeability towards water-selective membranes.

In this study, three different imidazolium-based ionic liquids containing alkyl chains with varying length and counter anions bis(trifluoromethanesulfonyl)imide ([TFSI]^−^) or tricyanomethanide ([C(CN_3_)]^-^) were incorporated into an aromatic polyether bearing side PEO units (2,6Py-PEO_350_) to yield free-standing, soft composite membranes. The effect of IL nature and loading on morphological, thermal and gas transport properties of the prepared 2,6Py-PEO_350_/IL composite membranes were studied. The water vapor transport properties were also evaluated.

## 2. Materials and Methods

### 2.1. Materials

Dimethylacetamide (DMAc, 99%) was supplied by Sigma-Aldrich (Burlington, MA, USA). Lithium bis(trifluoromethane)sulfonamide (LiTFSI, 99%), 1-ethyl-3-methylimidazolium chloride ([EMIM][Cl], 98%), 1-ethyl-3-methylimidazolium tricyanomethanide ([EMIM][C(CN)_3_], 98%) and 1-hexyl-3-methylimidazolium bis(trifluoromethane)sulfonamide ([HMIM] [TFSI], 99%) were purchased from IoLiTec (Heilbronn, Germany). All chemicals were used without further purification. Carbon dioxide (CO_2_), methane (CH_4_), hydrogen (H_2_) and helium (He) with a 99.999% level of minimum purity (for CH_4_, 99.99%) were provided by Air Liquide Hellas (Athens, Greece).

### 2.2. IL Preparation

For the preparation of 1-ethyl-3-methylimidazolium bis(trifluoromethane)sulfonamide ([EMIM][TFSI]), 1 g (6.82 mmol) of [EMIM][Cl] was dissolved in 6 mL of acetonitrile, and a solution of LiTFSI (2.34 g, 8.184 mmol) in 6 mL of acetonitrile was also prepared. Then, the LiTFSI solution was added dropwise to the IL solution at room temperature and left under stirring for 2 days to achieve maximum yield of anion exchange. The mixture was filtered and washed with acetonitrile and methanol for the removal of the excess LiTFSI salt. Finally, the obtained IL was recrystallized in dichloromethane for further purification.

### 2.3. Synthesis of Precursor Copolymer (2,6Py-PEO_350_)

**Scheme 1:**
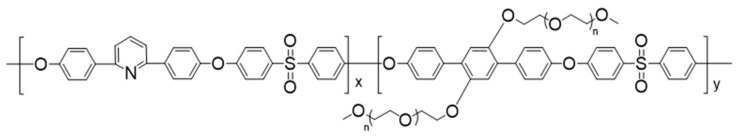
The precursor copolymer 2,6Py-PEO_350_ with composition x = 60 and y = 40.

The synthesis of 2,6Py-PEO_350_ copolymer was carried out according to our previous work [8]. In particular, a polycondensation reaction was used to produce aromatic polyethers containing main chain 2,6-substituted pyridine segments and short side chain PEO groups (MW = 350, ethylene oxide repeating units) with a composition of 60/40 (Scheme 1) corresponding to a 39 wt% PEO content. This copolymer exhibits excellent film-forming ability, owing to its high molecular weights (Mn = 36,400, Mw = 57,000, PDI = 2.2).

### 2.4. Membrane Preparation

**Scheme 2:**
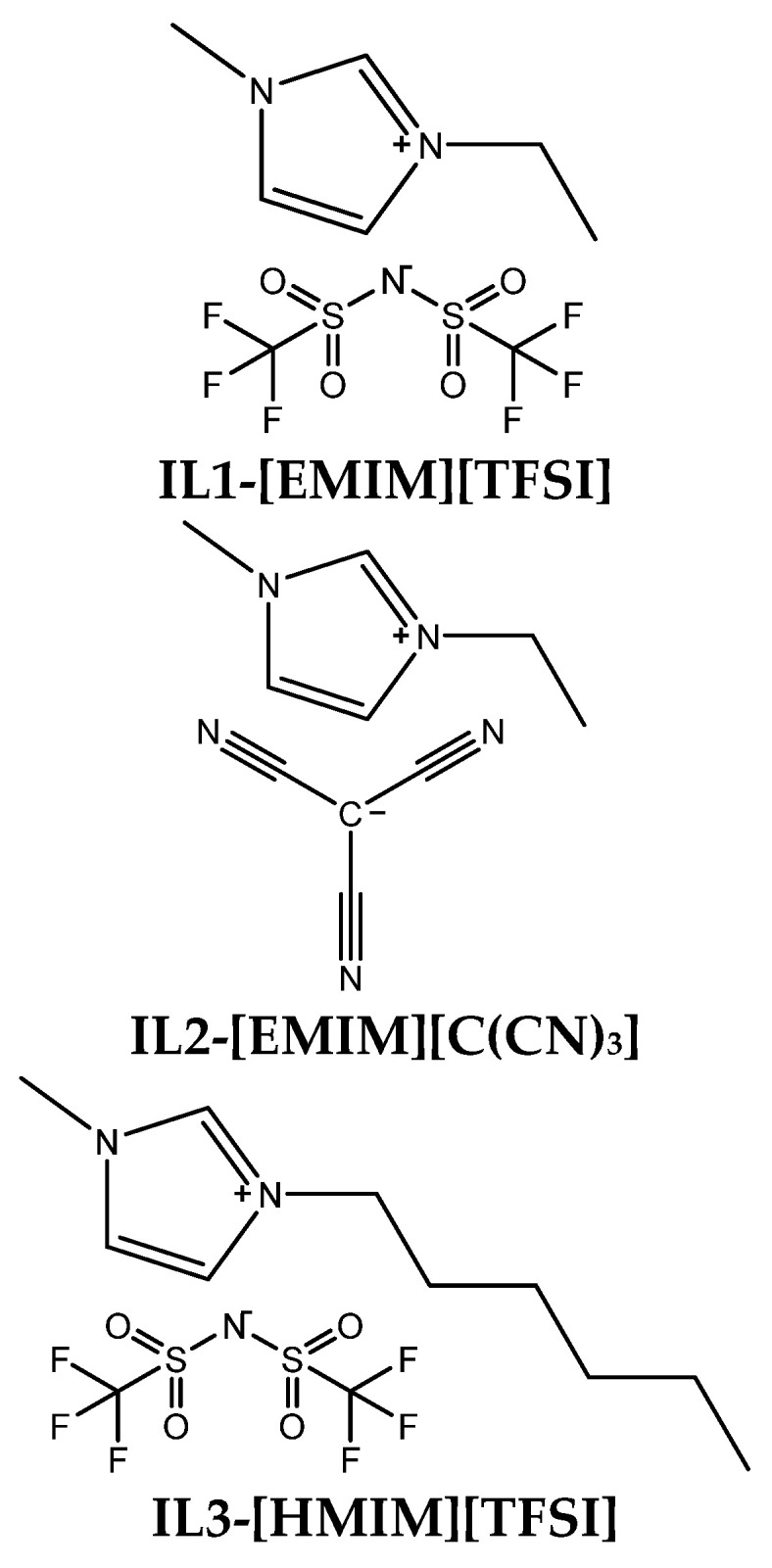
Chemical structures of the ionic liquids used to prepare polymer-IL composite membranes.

All composite and precursor membranes were prepared using the solution-casting method. First, the polymer solution was prepared by dissolving 200 mg of 2,6Py-PEO_350_ polymer in DMAc (5 wt% concentration) at room temperature. Different amounts of three different ionic liquids (IL1-[EMIM][TFSI], IL2-[EMIM][C(CN)_3_] and IL3-[HMIM][TFSI]) were added to the polymer solution under stirring to form a homogeneous mixture. Then, it was poured onto a flat glass plate and the solvent was slowly evaporated at 80 °C for 24 h. All prepared membranes were dried under vacuum at 80 °C until a constant weight was achieved. Polymer-IL composite membranes are hereafter referred to as 2,6Py-PEO_350_-xILz, where x denotes the wt% concentration of IL used, and z defines the kind of IL used, as shown in Scheme 2.

### 2.5. Membrane Characterization

ATR spectra of all membranes were collected from 4000 to 400 cm^−1^ with a resolution of 4 cm^−1^ using a platinum ATR Bruker spectrometer. Thermogravimetric analysis (TGA) was performed on a Labys TG (Setaram Instrumentation, Caluire-et-Cuire, France) under N_2_ flow from 25 to 800 °C with a heating rate of 10 °C min^−1^. Differential scanning calorimetry (DSC) was conducted on a Perkin Elmer DSC Q100 instrument (Waltham, MA, USA) under N_2_ flow, in the temperature range from −100 °C to 200 °C and with a heating rate of 10 °C min^−1^. Two heating cycles were performed, and the glass transition temperature was derived from the second cycle. Scanning electron microscopy (SEM) was conducted using a LEO Supra 35VP microscope (Zeiss, Oberkochen, Germany). The cross-sections of the membranes were prepared after fracturing in liquid N_2_.

### 2.6. Single Gas and Gas/Water Vapor Permeation Properties

Single gas permeation properties of precursor 2,6Py-PEO_350_ and composite membranes were studied at 30 °C using the Wicke–Kallenbach method. The experimental procedure and the description of the permeation cell were previously reported [33]. The addition of 3% H_2_O in each single gas at the retentate side was only studied for the 2,6Py-PEO_350_-40IL3 composite membrane, which showed the best single gas permeation properties. While He flow rate of 20 cm^3^ min^−1^ was employed in the permeate side for measurements under dry conditions, He flow rate of 100 cm^3^ min^−1^ was used for wet conditions. The gas permeability (*P*) was calculated according to the equation:

(1) $$P=\frac{\varphi_{v,tot}\times F}{A}\times \frac{l}{p_{r}-p_{p}}\;$$

where *A* is the effective membrane area, *p_r_* and *p_p_* are the retentate and permeate side partial pressures of the measured gas, respectively, *F* is the volume fraction of the measured gas present in helium flow, *l* is the membrane thickness and *φ_v,tot_* is the volumetric flow rate of helium at the permeate side.

Selectivity (*α_A/B_*) was calculated as the ratio of the permeabilities *P_A_* and *P_B_* of the gases A and B, respectively, using the following equation:

(2) $$a_{A/B}=\frac{P_{A}}{P_{B}}$$

## 3. Results and Discussion

### 3.1. Preparation and Characterization of Polymer-IL Composite Membranes

**Figure 1:**
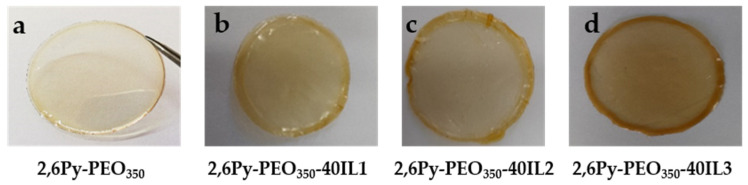
Pictures of the prepared neat polymer (**a**) and polymer-IL composite membranes with (**b**) 40 wt% of IL1, (**c**) 40 wt% of IL2 and (**d**) 40 wt% of IL3.

Mechanically robust, amorphous, soft PEO-based membranes with high PEO contents based on aromatic polyether copolymers bearing main chain 2,6-substituted pyridine segments and short side chain PEO groups (2,6Py-PEO_350_) have recently been prepared and investigated as CO_2_ and water-selective membranes by our group [8]. The high content of CO_2_-philic PEO groups induces severe plasticization of the rigid polymer backbone, as reflected by the shifting of T_g_ values to lower temperatures (close to room temperature), thereby resulting in increased chain mobility and flexibility, which in turn leads to increased gas/vapor diffusion. In addition, it has been proven that the incorporation of ionic liquids into the polymer matrix can further improve CO_2_ permeability based on their ionic character. For this reason, 2,6Py-PEO_350_ copolymer containing 39 wt% PEO was blended with ionic liquids (30 and 40 wt% contents) to prepare composite membranes aiming at CO_2_ permeability and separation performance enhancement. In this case, imidazolium-based ILs including IL1-[EMIM][TFSI], IL2-[EMIM][C(CN)_3_] and IL3-[HMIM][TFSI] were chosen, owing to their high CO_2_ solubility [16,18]. All the fabricated composite membranes were flexible, self-standing and soft, as illustrated in Figure 1. It should be noticed that the fabrication of free-standing films with IL loading higher than 40 wt% was not possible because of the instability of the resulting membranes.

**Figure 2:**
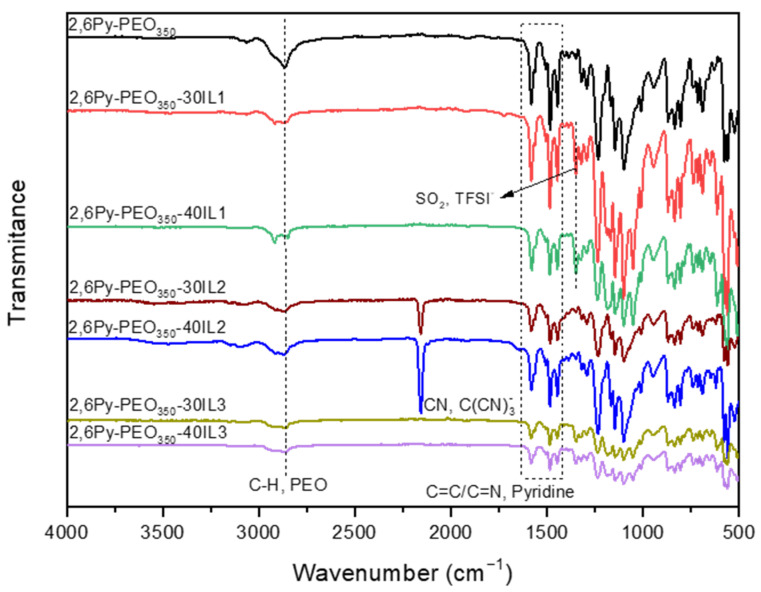
ATR-FTIR spectra of the precursor polymer and the polymer-IL composite membranes.

As illustrated in Figure 2, the precursor copolymer 2,6Py-PEO_350_ membrane along with the polymer-IL composite membranes were studied by ATR-FTIR spectroscopy. Regarding the precursor spectrum, the peak signal at 2868 cm^−1^ is assigned to methylene group vibrations of the PEO units [8,34]. In addition, the doublet peak at 1487 and 1471 cm^−1^ and the peak signal at 1583 cm^−1^ correspond to the C=C/C=N vibrations of the pyridine ring [8,35]. Considering that all ILs used in this work for the preparation of polymer-IL composite membranes contain a common imidazolium cation, the characteristic peak at 1574 cm^−1^ assigned to the imidazolium ring in plane symmetric/asymmetric stretch and –CH_2_(N)/-CH_3_(N)CN could not be observed in all composite membrane spectra due to overlapping with the peak at 1583 cm^−1^. However, after the addition of ILs containing a TFSI^-^ anion, a new peak at 1350 cm^−1^ appears that can be attributed to the asymmetric stretching of the SO_2_ group [9]. The symmetric bending vibrations of the same group are located at 616 cm^−1^ and 604 cm^−1^. The peak located at 1053 cm^−1^ is assigned to the asymmetric stretching band of the S–N–S group, and its bending vibrations can be identified as very weak peaks at 650 cm^−1^ and 612 cm^−1^. The bands at 789 cm^−1^ and 509 cm^−1^ are also attributed to asymmetric bending of the CF_3_ group and the C-S bond of the TFSI^-^ anion, respectively. After addition of IL containing a C(CN)_3_^-^ anion, a new strong peak arose at 2158 cm^−1^, attributed to the stretching vibration of the -CN group [9,36]. No shifts were observed in the ATR-IR spectra of 2,6Py-PEO_350_-IL membranes compared to those of neat 2,6Py-PEO_350_ copolymers.

**Figure 3:**
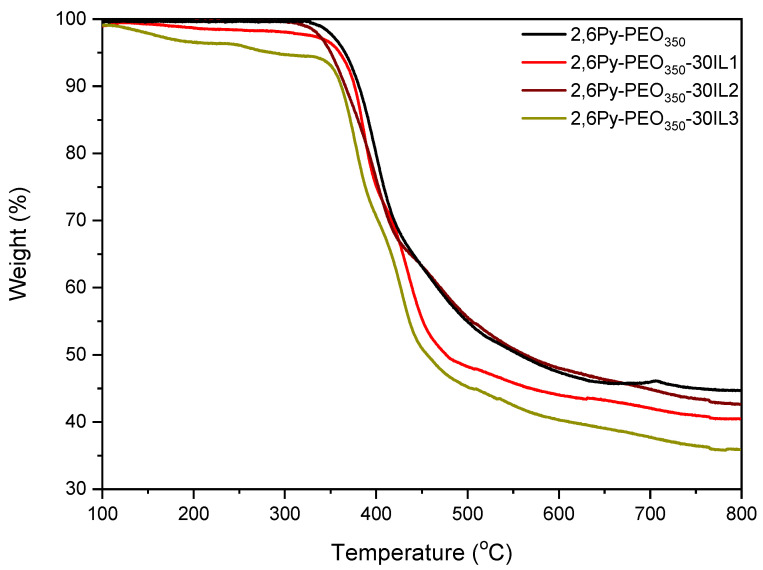
Comparative TGA curves of precursor-PEO_350_ and polymer composite membranes with 30 wt% IL.

Thermogravimetric analysis (TGA) was used to investigate the thermal stability of polymer-IL composite membranes. The TGA curves of precursor polymer and composite membranes containing 30 wt% IL are given in Figure 3. The precursor shows two main degradation steps, where the first step is observed at around 330 °C, assigned to the loss of PEO side groups [8] and the second one is at 430 °C, corresponding to the polymer backbone degradation. Regarding composite membranes containing IL1-[EMIM][TFSI] and IL3-[HMIM][TFSI], an initial weight loss up to 210 °C (1.5 and 3.5 wt%, respectively) was observed, attributed to DMAc residual solvent. For the latter, a second small weight loss at around 320 °C was evident, attributed to the evaporation of the corresponding IL3 [37]. All composite membranes show two main weight loss steps. The first starts at around 305–330 °C, associated with the degradation of side PEO groups, and the second one, at about 400–430 °C, is due to polymer backbone and ionic liquid degradation simultaneously. Indeed, for example, the reported degradation temperature of IL1 is 393 °C, which is in good agreement with our results [38]. Comparison of the TGA curves of the composite membranes containing imidazolium ILs with different alkyl chain length reveals that thermal degradation of the composite membrane with an imidazolium cation with a shorter alkyl chain length (IL1) starts at a higher temperature (T_d_ ~ 330 °C) than the degradation of the corresponding with the longer alkyl chain (T_d_ ~ 320 °C). This result may be explained by the stronger electrostatic interactions between the precursor copolymer and ionic liquid containing imidazolium cations with a shorter alkyl chain length, thus delaying the composite decomposition. In addition, the effect of anion nature on thermal stability was also investigated. Among composite membranes containing 30 wt% IL, the one with the TFSI^-^ anion shows higher thermal stability than the corresponding with the C(CN)_3_^-^ anion (the decomposition for the former starts at 330 °C, and for the latter at 305 °C). This is in agreement with the degradation temperatures determined for neat ILs [38]. The thermal stability of all prepared membranes incorporated with ionic liquid was found to be sufficient at temperatures up to ~300 °C, suggesting the suitability of these membranes for gas separation applications.

**Figure 4:**
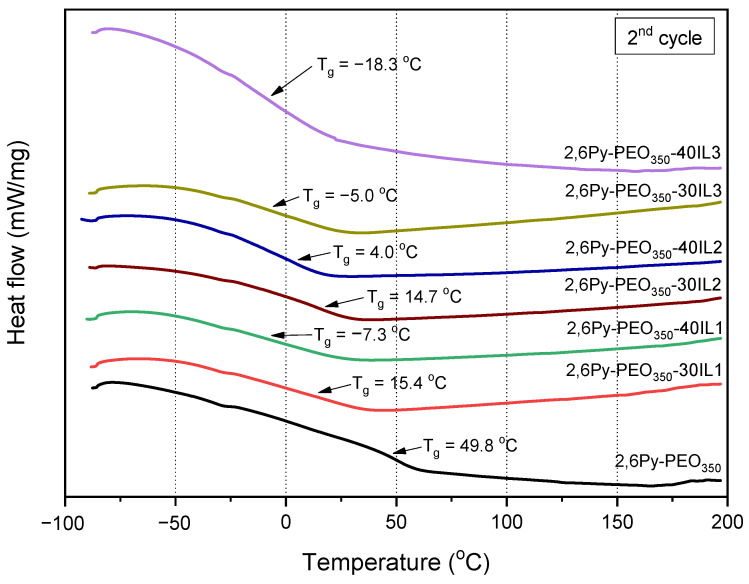
DSC curves for the precursor polymer and the polymer-IL composite membranes.

Differential scanning calorimetry (DSC) was used to determine the glass transition temperatures of the polymer-IL composite membranes, as depicted in Figure 4. Generally, all composite membranes display lower glass transition temperatures (T_g_s) compared to the neat copolymer, with a more pronounced reduction for higher IL contents. For instance, the composite membrane containing 30 wt% IL1-[EMIM][TFSI] has a T_g_ value of 15.4 °C, while an increase in IL1 content (40 wt%) is followed by a further T_g_ decrease to −7.3 °C. This is due to the plasticization effect, resulting in flexible and soft membrane structures [18] with increased free volume and, consequently, lower T_g_, as already reported [35,38]. Regarding the effect of the anion’s chemical structure on T_g_, the comparison of composite membranes containing the same IL content (40 wt%) but ILs with different counter anions (TFSI^-^ and C(CN)_3_^-^), reveals that the T_g_ of composite membranes containing IL2-[EMIM][C(CN)_3_] is higher (4 °C) than the corresponding of composite membranes with IL1. This can probably be attributed to the lower molar volume of the C(CN)_3_^-^ anion (123.33 Å^3^) [39] compared to the TFSI^-^ anion (208.3 Å^3^) [40]. As a consequence, the chain mobility is restricted since the C(CN)_3_^-^ anion can be accommodated in the available free interchain space, thus leading to the free volume reduction and, thereby, to T_g_ increase. The lowest T_g_ value of −18.3 °C was observed for the composite membrane comprising an imidazolium cation with hexyl groups (IL3) having 40 wt% IL content, while the corresponding one with shorter chain length (ethyl groups) has a T_g_ value of −7.3 °C. The increase of the alkyl chain length of the cation results in free volume and chain mobility increase, thus leading to T_g_ decrease. For all composite membranes, the presence of a single T_g_ indicates the successful incorporation of the three different ILs into the polymer matrix. It should be noted that all composite membranes are in the rubbery state at 30 °C, the temperature used for water vapor and gas permeation experiments in this study.

**Figure 5:**
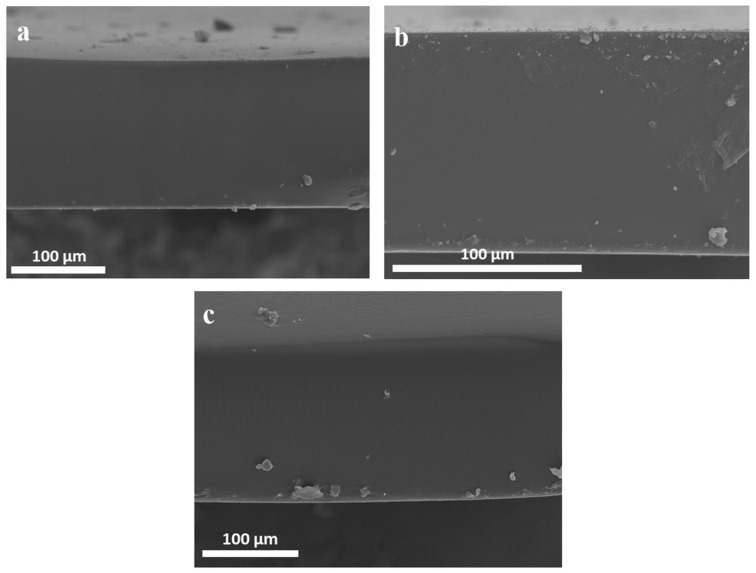
SEM micrographs of composite membranes containing 40 wt%. (**a**) [EMIM][TFSI], (**b**) [EMIM][C(CN)_3_] and (**c**) [HMIM][TFSI].

The membrane morphology plays a vital role in gas separation and purification performance. The cross-sectional SEM micrographs of polymer-IL composite membranes containing 40 wt% of each IL are illustrated in Figure 5. It was revealed that all membranes are dense, smooth and not phase-separated. This suggests that ILs were uniformly distributed into the polymer matrix, leading to non-porous, homogeneous structures. Similar observations were reported for the Pebax^®^1657/(Emim]BF_4_]) polymer IL pair by Rabiee et al. [18]. The thickness of the membranes ranges from 47 to 165 μm.

### 3.2. Gas Separation Properties of Polymer-IL Composite Membranes

The gas separation properties of the 2,6Py-PEO_350_ precursor copolymer and the composite membranes were determined using the Wicke–Kallenbach method at 30 °C. The gas permeability data (CO_2_, CH_4_, H_2_) are shown in Table 1. It was observed that the precursor copolymer exhibits much lower gas permeability than composite membranes containing the imidazolium-based ILs with TFSI^-^ (IL1 and IL3). Generally, IL molecules plasticize the polymer matrix, leading to improved chain mobility and increased free volume, and consequently to enhanced gas diffusivity and permeability [18,41,42,43,44]. However, solubility also plays a significant role in permeability according to the solubility-diffusion mechanism for dense membranes [45]. For instance, ILs with TFSI^-^ and C(CN)_3_^-^ as counter anions are reported to show high CO_2_ solubility due to chemical interactions of CO_2_ molecules with the anions [46,47,48]. In addition, the higher fractional free volume of ILs compared to polymers facilitates the CO_2_ diffusion across composite membranes [38]. In contrast to this, CH_4_ and H_2_ have no significant interactions with ILs [38], thus very small additional solubility contribution is expected for CH_4_ and H_2_ in composite membranes, and, therefore, diffusion mainly contributes to CH_4_ and H_2_ permeability increases. As a result, the permeability increment is more significant for CO_2_ instead of H_2_ and CH_4_ due to the high diffusivity and solubility of CO_2_ in a polymer and IL matrix, respectively. For this reason, CO_2_/CH_4_ and CO_2_/H_2_ ideal selectivities are also increased with the addition of ILs. In contrast, the incorporation of the imidazolium-based IL with C(CN)_3_^-^ (IL2) into the polymer matrix caused a clear reduction of CH_4_ and H_2_ permeability, while CO_2_ permeability was almost not affected. The reduction is more pronounced for H_2_ than CH_4_. This phenomenon could be possibly attributed to two reasons. The lower molar volume of the C(CN)_3_^-^ counter anion enables IL accommodation in the initial available free interchain spacing, thus restricting chain mobility and resulting in smaller interchain spacing for gas transport. Another reason could be the strong interactions of the IL cation with the C(CN)_3_^-^ anion and/or PEO groups of the precursor with IL ionic moieties that can lead to a tighter and more compact structure. As the restriction induced by the strong interactions has a more considerable effect on permeability than plasticization, the CH_4_ and H_2_ permeabilities were decreased.

The effect of IL content on gas separation properties was studied. The permeability of all gases is significantly increased by increasing the IL loading in the host polymer matrix for all composite membranes. Besides plasticization effect and free volume increase discussed above, the higher intrinsic permeability of ILs may also contribute significantly to the increase of gas permeabilities [46]. For instance, 2,6Py-PEO_350_ containing 40 wt% [EMIM][TFSI] (IL1) exhibited almost 40% higher gas permeabilities than the corresponding membrane with IL loading 30 wt%. In specific, CO_2_ permeability is increased from 17.3 Barrer to 24.7 Barrer by increasing the IL1 content from 30 to 40 wt%, while ideal selectivity of CO_2_/CH_4_ remains constant. The same trend is also reported for systems including Pebax^®^1657/[BMIM][CF_3_SO_3_] and PolyActive^TM^/[EMIM][BF_4_] composite membranes [18,19]. The positive effect of IL content on CO_2_ permeability and CO_2_/CH_4_ selectivity was observed for glassy polyether sulfone/[EMIM][TFSI] membranes as well, where CO_2_ permeability increased from 196.11 Barrer to 255.30 Barrer with increasing IL content from 30 to 40 wt% [38].

Another factor that may influence the gas separation properties is the alkyl chain length in the imidazolium cation. As shown in Table 1, by comparing the CO_2_ and CH_4_ permeability values of 2,6Py-PEO_350_-40IL3 containing the hexyl chain substituent in the cation with the corresponding membrane having the ethyl chain substituent (IL1), it is evident that the former has 1.9- and 2.5-times higher values, respectively, than the latter. The longer alkyl chains probably enable the better distribution of the IL into the polymer matrix, thus inducing a stronger plasticization and free volume increase, which facilitates gas diffusion and, consequently, gas permeability increase. The same trend was also reported by X. Li et al. [23]. In particular, Pebax^®^1657 composite membranes containing imidazolium ILs having different alkyl chain lengths (ethyl and hexyl groups in the cation) were prepared, and a significant CO_2_ permeability increment was observed with increasing alkyl chain length.

Concerning the effect of the IL anion on gas transport, it was observed that composite membranes containing the C(CN)_3_^-^ anion (IL2) exhibit significantly lower permeability for all gases than the corresponding membranes with the TFSI^-^ anion (with 30 or 40 wt% IL content). This is attributed to the denser polymer chain packing due to strong interactions of IL cation and anion and/or PEO groups with IL ionic moieties, thus contributing to gas diffusion decrease and, thereby, to gas permeability decrease [9]. However, in supported IL membranes containing TFSI^-^ and C(CN)_3_^-^ anions, the CO_2_ permeability appears not to be affected by the type of anion under the same measurement conditions [49]. On the other hand, it would be expected that, due to the high number of cyano groups contained in IL2, CO_2_ permeability and solubility should be high [48]. However, this seems not to be the case neither for the prepared composite membranes of this work as evidenced by the gas permeability data, nor for other PEO-based PILs [9].

Pure gas permeabilities of the precursor and the polymer-IL composite membranes follow the order CO_2_ (3.30 Å) > H_2_ (2.87 Å) > CH_4_ (3.80 Å) [50], where CO_2_ has higher permeability than H_2_, although the latter has smaller kinetic diameter. Consequently, the precursor and all composite membranes exhibit CO_2_/H_2_ “reverse selectivity”, in which CO_2_ is preferentially transported across the membrane over H_2_. This is mostly characteristic for rubbery polymers or those that can strongly interact with CO_2_ molecules such as PEO-based membranes [8]. In specific, these polymers exhibit high CO_2_ solubility due to significant interactions of quadrupole CO_2_ molecules with polar PEO groups, thus resulting in higher CO_2_ permeability. In glassy polymers, the opposite trend is observed, as the diffusion-controlled gas transport dominates leading to the preferential transport of H_2_ over CO_2_. The composite membrane with 40 wt% [HMIM][TFSI] (IL3) achieved the highest CO_2_ gas permeability value of 46.1 Barrer with corresponding ideal selectivity values of 39 and 5.6 for CO_2_/CH_4_ and CO_2_/H_2_ pairs, respectively.

**Figure 6:**
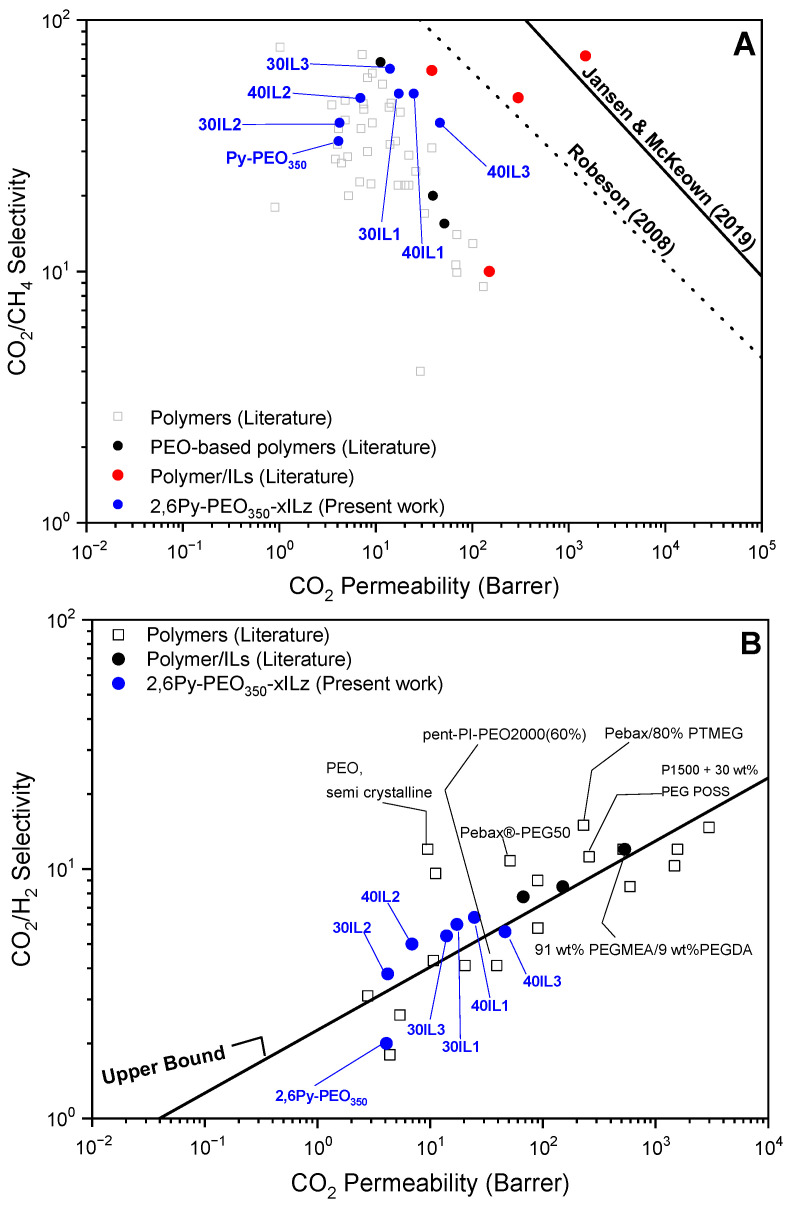
Permeability–selectivity upper bound plots of 2,6Py-PEO_350_ and composite membranes (blue circles) for (**A**) CO_2_/CH_4_ and (**B**) CO_2_/H_2_ selectivity. The 2008 and 2019 upper bounds for CO_2_/CH_4_ (35 °C) were adapted from refs. [22,51]. The upper bound for CO_2_/H_2_ (25 °C) was adapted from refs. [52,53]. The rest of the data points were taken from refs. [7,52,54,55,56,57,58,59,60,61,62,63,64].

Comparison of gas permeability and selectivity of the precursor and the prepared composite membranes for the CO_2_/CH_4_ gas pair with a PEO-based polymer and other polymer/IL membranes reported in the literature is shown in Figure 6A. It can be noticed that their separation performance is comparable or even higher than the other membranes investigated. In addition, all prepared composite membranes show improved CO_2_/CH_4_ separation performance (selectivity varying in the range of 39–64) compared to the precursor membrane (33), owing to the presence of ILs, which exhibit high CO_2_ solubility and permeability. These data lie close but below the 2008 Robeson upper bound line. The composite membrane containing 30 wt% IL3 exhibits the highest CO_2_/CH_4_ selectivity (64) among all composite membranes. The significant increase of CO_2_/CH_4_ selectivity (almost 2 times higher than the precursor membrane) can be ascribed to the relative permeability increment of CO_2_, which is more pronounced compared to that of CH_4_. While for methane the permeability increase is mostly owed to diffusivity increase, the contribution of both solubility and diffusivity on permeability enhancement for CO_2_ is evident. A similar CO_2_/CH_4_ selectivity value of 72 for Pebax^®^MH 1657 blended with 20 wt% of [C_4_MIM][Gly] was reported [23]. Although with increasing IL content there is no clear trend for CO_2_/CH_4_ selectivity, in the case of composite membranes containing IL2, both CO_2_ permeability and CO_2_/CH_4_ selectivity increased, suggesting that this system does not follow the typical “trade off” behavior between permeability and selectivity. On the contrary, the increase of IL content in composite membranes containing IL3 results in the deterioration of CO_2_/CH_4_ separation performance. In addition, the anion’s effect on CO_2_/CH_4_ selectivity was studied and revealed that by switching from TFSI^-^- (IL3) to C(CN)_3_^-^-containing composite membranes, the selectivity is reduced from 64 to 39, respectively. This is mainly related to the reduction of CO_2_ permeability, which is more significant compared to that of CH_4_. A possible explanation is that composite membranes containing the C(CN)_3_^-^ counter anion exhibit a tighter polymer chain packing due to strong interchain electrostatic interactions between the IL cation and anion and/or between PEO groups and IL ionic moieties, which hinders gas diffusion and thus leads to permeability decrease.

As evidenced in Figure 6B, almost all polymer-IL composite membrane surpassed the upper bound limit for CO_2_/H_2_ separation, while precursor polymer 2,6Py-PEO_350_ lies below this limit. All composite membranes (with 30 wt% IL) showed simultaneously higher CO_2_ permeability and CO_2_/H_2_ selectivity than the neat copolymer. The CO_2_/H_2_ ideal selectivity enhancement can be mainly attributed to the increased CO_2_ diffusivity and CO_2_ solubility of the ILs. By increasing IL content from 30 to 40 wt%, the CO_2_/H_2_ selectivity was further increased. The positive effect of increasing IL content on CO_2_/H_2_ selectivity was also reported for Pebax^®^1657/[Emim][BF_4_] [18]. The 2,6Py-PEO_350_ with 40 wt% [EMIM][TFSI] (IL1) has the best CO_2_/H_2_ selectivity value of 6.4 and a CO_2_ permeability value of 24.7 Barrer. The separation performance of the composite membranes of this work is comparable with other PEO-based copolymer membranes and polymer/IL composite membranes reported in the literature, despite their lower CO_2_ permeability, as depicted in Figure 6B. For instance, Bernardo et al. studied the separation performance of Pebax^®^1657 blended with 20–80 wt% [BMIM][CF_3_SO_3_] and reported that the composite membrane containing 40 wt% IL content exhibited an ideal CO_2_/H_2_ selectivity value of 8.5 and a CO_2_ permeability value of 150 Barrer [12].

In conclusion, a prospective strategy for enhancing the CO_2_ separation properties of polymer-IL composite membranes comprises the successful tailoring of IL types (cation, anion) having high CO_2_ solubility combined with a high CO_2_ affinity polymer matrix.

### 3.3. Water Vapor Separation Properties of Polymer-IL Composite Membranes

**Figure 7:**
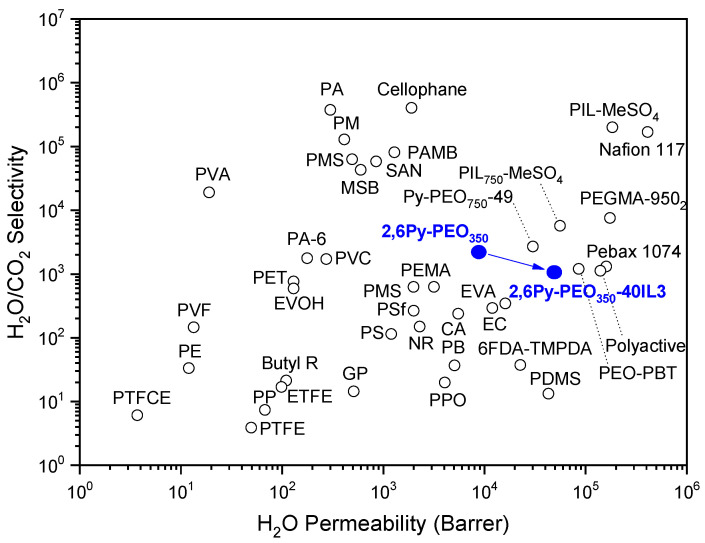
Comparison of H_2_O permeability with H_2_O/CO_2_ selectivity of 2,6Py-PEO_350_ and 2,6Py-PEO_350_-40IL3 membranes (blue circles) with other polymers reported in the literature (25–35 °C) [33].

The water vapor permeation properties of the precursor polymer and the 2,6Py-PEO_350_-40IL3 composite membrane with the highest gas permeabilities (CO_2_, CH_4_, H_2_), were also studied (Figure 7, Table 2). In specific, the precursor polymer had a water vapor permeability of 8900 Barrer, as the presence of polar pyridine units in the polyether backbone and side hydrophilic PEO groups enables the formation of hydrogen bonds with water, thus promoting water vapor permeability. The addition of 40 wt% [HMIM][TFSI] (IL3) into the polymer matrix had a positive effect on water vapor permeability. In specific, a substantial increase of water vapor permeability by 5.6 times to 50,000 Barrer was observed. The strong plasticization induced by the incorporation of IL3 causes a significant increase of free volume, thus facilitating water vapor diffusion and, consequently, water vapor permeability enhancement. It should be stressed that water vapor permeability is mainly governed by the hydrophilicity and hydrogen bond basicity of the counter anion in PILs, as recently reported by our group [9]. In the case of the 2,6Py-PEO_350_-40IL3 composite membrane, despite the hydrophobic character of [HMIM][TFSI] (IL3) and its weak proton acceptor ability (due to the electron-withdrawing effect of fluorine atoms in TFSI^-^), the hydrogen bond between water molecules and the TFSI^-^ anion do form. However, the water vapor permeability improvement is mainly attributed to the high diffusivity and to a lesser extent to solubility. The H_2_O/CO_2_ selectivity of the composite membrane was significantly declined by 50% to 1080 compared to the corresponding one of precursor membrane. To the best of our knowledge, water vapor permeability data of polymer-IL composite membranes are limited, and, consequently, a comparison can be made only with hydrophilic polymers containing PEO groups. As illustrated in Figure 7, the water vapor permeation behavior of the present composite membrane is on par with other PEO-based polymers such as Pebax^®^1074, Polyactive^TM^ and PEO-PBT. These results underline the potential of this composite membrane for dehydration applications.

Gas permeation properties of the 2,6Py-PEO_350_-40IL3 membrane were also studied under wet conditions. A total of 3% H_2_O was added in the feed flow of each gas at the retentate side to investigate how the presence of water can affect gas permeability and selectivity. The gas permeation properties of 2,6Py-PEO_350_-40IL3 under dry and wet conditions are given in Table 2. It is evident that CO_2_ and CH_4_ permeability remained unchanged (considering the given errors), while H_2_ permeability was slightly increased (11.0 Barrer). As a result, gas selectivities/separation factors (SF) were not noticeably affected, and H_2_O/gas separation factors remained unaltered. On the other hand, Dai et al. [65] used the IL [TETA][tfa] for the preparation of Pebax^®^/TSIL blends and reported that the presence of water vapor (high relative humidity) in feed gas enables membrane swelling, which favors gas diffusion. In specific, CO_2_ permeability of a composite membrane containing 30 wt% IL changed from around 250 to 567 GPU, denoting the positive role of water vapor on gas transport through membranes. As [TETA][tfa] is a very hydrophilic IL, the membrane is strongly plasticized by water molecules, thus leading to gas diffusion increase. In contrast, in the case of the prepared membrane containing the [HMIM][TFSI], swelling is not favored due to the IL’s hydrophobic character. For this reason, the presence of water vapor does not affect the separation properties.

## 4. Conclusions

Free-standing PEO-based copolymer-IL composite membranes were successfully prepared via the solution casting method by incorporating up to 40 wt% of three different ILs into the 2,6Py-PEO_350_ polymer matrix. The prepared composite membranes are amorphous and rubbery with low T_g_ values, as revealed by DSC. In addition, they are homogeneous, indicative of the good dispersion of the ILs into the polymer matrix and have high thermal stability up to ~300 °C, as evidenced by SEM and TGA analysis, respectively. The addition of ILs containing different alkyl chain length in imidazolium and anions (TFSI^-^, C(CN_3_)^-^) into the polymer matrix (30 wt%) caused a significant increase of both CO_2_ and CO_2_/H_2_ selectivity compared to the neat PEO-based copolymer. The composite membrane with 40 wt% [HMIM][TFSI] (IL3) achieved the highest CO_2_ gas permeability value of 46.1 Barrer with corresponding ideal selectivity values of 39 and 5.6 for CO_2_/CH_4_ and CO_2_/H_2_ pairs, respectively. Almost all polymer-IL composite membranes surpassed the upper bound limit for CO_2_/H_2_ separation, while the precursor polymer 2,6Py-PEO_350_ lies below this limit. The CO_2_/H_2_ ideal selectivity enhancement can be mainly attributed to the increased CO_2_ diffusivity and CO_2_ solubility of the ILs. Regarding the alkyl chain length in the imidazolium cation, the comparison of the CO_2_ and CH_4_ permeability values of 2,6Py-PEO_350_-40IL3 containing the hexyl chain substituent in the cation with the corresponding membrane having the ethyl chain substituent (IL1) revealed that longer alkyl chains lead to free volume increase, which facilitates gas diffusion and, consequently, gas permeability increase. The positive effect of increasing IL loading on gas permeabilities was also revealed. Concerning the IL anion’s nature effect on gas transport, it was observed that composite membranes containing the C(CN)_3_^-^ anion (IL2) exhibit significantly lower permeability for all gases than the corresponding membranes with the TFSI^-^ anion (with 30 or 40 wt% IL content), which is attributed to the denser polymer chain packing that hinders gas diffusion and therefore results in reduced permeability. The composite membrane with 40 wt% [HMIM][TFSI] (IL3) also exhibits the highest water vapor permeability value of 50,000 Barrer and a corresponding H_2_O/CO_2_ ideal selectivity value of 1080, which are comparable to those reported for other highly water-selective PEO-based polymers. Therefore, it could be a potential candidate for hydrogen purification and dehydration of CO_2_ gas streams.

## Figures and Tables

**Table 1:** Single gas permeability and CO_2_ selectivity over other gases of the precursor polymer and the polymer-IL composite membranes (1 Barrer = 10^−10^ cm^3^(STP) cm cm^−2^ s^−1^ cm Hg^−1^).

Polymer-IL Membranes	Membrane Thickness (μm)	CO_2_ Permeability (Barrer)	CH_4_ Permeability(Barrer)	H_2_ Permeability (Barrer)	CO_2_/CH_4_ Selectivity	CO_2_/H_2_ Selectivity
2,6Py-PEO_350_	50	4.1 ± 0.1	0.122 ± 0.003	2.0 ± 0.1	33 ± 1	2.0 ± 0.1
2,6Py-PEO_350_-30IL1	136	17.3 ± 0.4	0.340 ± 0.008	2.8 ± 0.9	51 ± 2	6 ± 2
2,6Py-PEO_350_-40IL1	161	24.7 ± 0.6	0.48 ± 0.01	3.9 ± 0.1	51 ± 2	6.4 ± 0.2
2,6Py-PEO_350_-30IL2	47	4.2 ± 0.1	0.11 ± 0.02	1.1 ± 0.1	39 ± 7	3.8 ± 0.5
2,6Py-PEO_350_-40IL2	119	6.9 ± 0.2	0.14 ± 0.03	1.5 ± 0.1	49 ± 9	5 ± 2
2,6Py-PEO_350_-30IL3	78	14.0 ± 0.3	0.220 ± 0.006	2.6 ± 0.1	64 ± 2	5.4 ± 0.3
2,6Py-PEO_350_-40IL3	165	46.1 ± 0.8	1.2 ± 0.1	8.2 ± 0.4	39 ± 3	5.6 ± 0.3

**Table 2:** Water vapor permeation properties of the precursor polymer and composite membrane containing 40 wt% [HMIM][TFSI] IL under dry and wet conditions (1 Barrer = 10^−10^ cm^3^(STP) cm cm^−2^ s^−1^ cm Hg^−1^).

Polymer-IL	Membrane Thickness (μm)	CO_2_ Permeability (Barrer)	CH_4_ Permeability (Barrer)	H_2_ Permeability (Barrer)	H_2_O Permeability (×10^4^ Barrer)	CO_2_/CH_4_ Selectivity	CO_2_/H_2_ Selectivity
2,6Py-PEO_350_ Dry conditions	50	4.1 ± 0.1	0.122 ± 0.003	2.0 ± 0.1	0.89 ± 0.02	33 ± 1	2.0 ± 0.1
2,6Py-PEO_350_-40IL3 Dry conditions	165	46.1 ± 0.8	1.2 ± 0.1	8.2 ± 0.4	5.0 ± 0.1	39 ± 3	5.6 ± 0.3
2,6Py-PEO_350_-40IL3 Wet conditions	166	52 ± 9	1.4 ± 0.1	11.0 ± 0.7	5.0 ± 0.1	37 ± 7	4.7 ± 0.9

## Data Availability

Not applicable.
